# Low Fischer’s ratio is associated with increased mortality in patients with kidney failure

**DOI:** 10.1038/s41598-026-46326-y

**Published:** 2026-04-06

**Authors:** Qianying Zhang, Mohamed E. Suliman, Abdul Rashid Qureshi, Fujia Guo, Dario Troise, Ricong Xu, Peter Bárány, Olof Heimbürger, Peter Stenvinkel, Bengt Lindholm

**Affiliations:** 1 Renal Medicine and Baxter Novum, Department of Clinical Sciences, Intervention and Technology, Karolinska University Hospital Huddinge, Karolinska Institutet, Stockholm, 141 86 Sweden; 2 Department of Nephrology, Ruijin Hospital, Shanghai Jiao Tong University School of Medicine, Shanghai, China; 3 First Affiliated Hospital, Dalian Medical University, Dalian, China; 4 Nephrology, Dialysis and Transplantation Unit, Advanced Research Center on Kidney Aging, Department of Medical and Surgical Sciences, University of Foggia, Foggia, Italy; 5 Department of Nephrology, The First Affiliated Hospital of Shenzhen University, Shenzhen, China

**Keywords:** Aromatic amino acids, Branched-chain amino acids, Fischer’s ratio, Inflammation, Protein energy wasting, Chronic kidney disease, Mortality, Biomarkers, Diseases, Medical research, Nephrology, Risk factors

## Abstract

Metabolic changes in kidney failure, including an altered ratio of plasma branched-chain amino acids (BCAAs) to aromatic amino acids (AAAs), also known as Fischer’s ratio (FR), could potentially affect survival. We analyzed associations of FR with mortality in patients with kidney failure. In 328 kidney failure patients starting dialysis (median age 54 years, 60% men), plasma concentrations of BCAAs (valine, isoleucine, and leucine) and AAAs (phenylalanine, tyrosine) were measured using HPLC. We used Fine-Gray competing-risk regression analysis with renal transplantation as competing risk to compare 5-year survival between low and middle tertiles versus high tertile of FR, and restricted cubic spline analysis to examine associations of BCAA, AAA and FR, as continuous variables, with mortality risk. During median 29.4 (range 0.7–60.0) months of follow-up, 169 patients (52%) underwent renal transplantation and 82 (25%) patients died. Compared to patients in the high FR tertile, those in the combined middle and low tertiles had higher cumulative incidence of mortality (sHR1.74, 95% CI 1.00–3.04, *p* = 0.04). FR was negatively correlated with inflammation markers and protein-energy wasting. Subgroup and interaction analyses showed that the association of FR with mortality was strongest among patients with cardiovascular disease (OR 4.10, 95% CI 1.53–11.00, *p* = 0.005; *p* for interaction = 0.009). A low Fischer’s ratio associated with increased mortality risk among individuals with kidney failure and its prognostic strength was most evident in patients with cardiovascular disease.

**Supplementary Information:**

The online version contains supplementary material available at 10.1038/s41598-026-46326-y.

## Introduction

Chronic kidney disease (CKD) is an escalating global health concern^1^ and is now among the major contributors to global disability-adjusted life-years^2^. The global prevalence of CKD varies between 7.2% and 14.0%, with higher rates in subgroups, particularly those with cardiovascular disease (CVD)^1,3–5^. Among CKD patients, approximately 0.8–1.4% progress to end-stage renal disease (ESRD), or CKD stage 5, characterized by a glomerular filtration rate (GFR) less than 15 ml/min, and requiring renal replacement therapy (RRT)^3,5^. Patients with ESRD face high risks of serious complications which may lead to disability and mortality^5^. Cardiovascular morbidity affects roughly 70% of dialysis population, and mortality in ESRD patients is 10–30 times higher than in the general population^5,6^. Among young adults with ESRD, the risk of CVD-related mortality increases more than 100-fold compared to age-matched individuals in the general population^7^.

Despite numerous efforts to identify risk factors and biomarkers predicting outcomes, and despite interventions targeting traditional risk factors and common comorbidities such as protein energy wasting (PEW), inflammation, anemia, CVD, hypertension, diabetes mellitus (DM), and dyslipidemia, the mortality rates in ESRD have remained relatively unchanged over the years^5–10^. Moreover, no single biomarker has been verified to be a reliable prognostic indicator for predicting outcomes in ESRD patients^11–15^. These observations may imply that ESRD represents a complex pathophysiological condition, driven by mechanisms not yet fully understood. This highlights the urgent need to explore additional risk factors and novel biological markers to improve our understanding and identify new therapeutic targets. One such potential target is amino acid metabolism.

Alterations in amino acids (AAs) profiles have been documented in various kidney diseases^16–19^. The kidneys are critically involved in the synthesis, metabolism and excretion of AAs^16,20,21^. Renal proximal tubules normally reabsorb nearly 99% of the AAs filtered by glomeruli. When GFR declines, disturbances in AAs balance occur. However, plasma levels of individual AAs vary in different directions, indicating that their dysregulation extends beyond impaired filtration and reabsorption or simple metabolite retention^22,23^. This suggests a broader role for altered metabolism of AAs in ESRD. Importantly, the complexity of AA interactions means that analyzing a single amino acid may not fully capture the metabolic changes occurring in ESRD.

One such measure is Fischer’s ratio (FR), i.e., the ratio of branched-chain amino acids (BCAAs) to two aromatic amino acids (AAAs), namely tyrosine (Tyr) and phenylalanine (Phe). Notably, this ratio does not include tryptophan among the AAAs. First introduced by Fischer in 1976^24,25^, FR was reported to predict outcomes in liver cirrhosis, heart failure and for detecting hepatocellular carcinoma in high-risk individuals^26–28^. However, to date, no study has evaluated the prognostic significance of FR in CKD patients. This study aimed to investigate the association between baseline FR and subsequent patient survival among CKD stage 5 patients starting dialysis.

## Materials and methods

### Study populations

We carried out a post hoc analysis of a cross-sectional study in a cohort of CKD stage 5 patients with follow-up data of mortality^29^. Patients were recruited between December 1994 and April 2014 and underwent baseline investigation in conjunction with start of maintenance dialysis treatment. Patients under 18 or over 70 years were excluded. Additional exclusion criteria included signs of overt infection, acute vasculitis, unwillingness to participate or missing data on amino acids needed for calculating FR.

A total of 560 patients were initially enrolled, of whom 328 patients (60.4% men) met the inclusion criteria for the current investigations. Their median age at enrollment was 54 years and the median estimated glomerular filtration rate (eGFR) was 6.3 ml/min per 1.73 m^2^. The primary diseases of CKD were diabetic nephropathy (26%), hypertension/renal vascular disease (21%), chronic glomerulonephritis (25%), and other causes (28%). Among them, 85% of patients were using antihypertensive drugs and 18% were on statins. Most patients received other therapies commonly used in kidney failure such as erythropoietin, phosphate binders and potassium binders.

Sixty-one (21%) patients received oral amino acid supplementation (Aminess N; Recip AB, Stockholm, Sweden) with tablets containing: histidine 45 mg, valine 135 mg, isoleucine 60 mg, leucine 90 mg, lysine 65 mg, methionine 90 mg, phenylalanine 70 mg, threonine 65 mg, tryptophan 25 mg, and tyrosine 75 mg.

Data on BCAAs and AAAs from 83 community-dwelling control subjects (median age 51 (range 21–80) years, 55 males) with similar age and sex distribution as the patients were used for comparative analyses. The control population is described in^30,31^.

Written informed consents were signed by each participant. The study protocol was approved by the Ethics Committee of the Karolinska Institute (EPN) at the Karolinska University Hospital Huddinge, Stockholm, Sweden. The study was conducted in adherence to the Declaration of Helsinki.

## Methods

Venous blood samples were collected in the morning after an overnight fast and, in those patients who had started dialysis, on a dialysis-free day. Plasma and serum were separated and stored at − 70^◦^C refrigerators if the samples were not analyzed immediately. The plasma concentrations of amino acids including BCAAs (leucine (Leu), isoleucine (Ile) and valine (Val)) and AAAs (phenylalanine (Phe) and tyrosine (Tyr)) were measured using reversed-phase high-performance liquid chromatography (HPLC) with fluorometric detection, as previously described^32^.

Serum cholesterol, triglycerides (TG) and high-density lipoprotein cholesterol (HDL-C) were measured using standard enzymatic procedures (Boehringer Mannheim, Mannheim, Germany). Nephelometry was used to determine serum high-sensitivity C-reactive protein (hsCRP). ELISA commercial kits (Roche Diagnostics GmbH, Penzberg, Germany) were used to measure serum interleukin-6 (IL-6). Other laboratory biochemical analyses were done with routine methods at Karolinska University Hospital at Huddinge. Estimated GFR (eGFR) was assessed from serum creatinine levels. The atherogenic index of plasma (AIP) was calculated as the logarithmically transformed ratio of the serum TG to HDL-c according to the following formula^33^: $\mathrm{AIP} = \mathrm{log}\frac{{TG}}{{HDL}-C}$. Subjective global assessment (SGA) of nutritional status was evaluated using the four-point SGA scale and protein energy wasting (PEW) was defined as SGA score > 1^34^. Body mass index (BMI) was calculated as body weight(kg) divided by the square of the subject’s height(m) (kg/m^2^).

In 229 (69.8%) of the patients, the baseline investigation was performed median (IQR) 21 (5–77) days before the start of dialysis and in 99 (30.2%) of the patients the baseline investigation was performed within median (IQR) 8(4-21) days after dialysis initiation.

Patients were followed for up to 5-years or until death or censoring. Survival time was calculated from the date of blood sampling to the date of death or censoring due to renal transplantation or end of follow-up period. No patient was lost to follow-up. The median follow-up period was 29.4 (range: 0.9–60) months. During the follow-up period, 82 patients (25%) died,169 patients (52%) underwent renal transplantation.

### Statistical analysis

Normally distributed data were presented as means (95% confidence interval (CI)), skewed distribution data were presented as medians (interquartile range (IQR)). Categorical data were presented as number and frequency (%). Non-parametric Wilcoxon test and Kruskal-Walli’s test were used for comparisons between two or more groups for continuous variables. Chi-square tests (*χ*^2^) or Fisher exact tests were applied for the comparison of nominal variables. Non-parametric Spearman rank correlation analysis was used to assess the associations between variables. Fine-Gray competing-risk regression analysis models with renal transplantation as a competing risk were used to compare the differences in survival rate between high tertile versus combined low and middle tertiles of FR and to estimate sub-distribution hazard ratio (sHR). Restricted cubic spline analysis was conducted to examine the associations of FR, concentrations of BCAA and AAA, as continuous variables, with the risk of all-cause mortality. Multivariate logistic regression analysis yielding odds ratios with 95% confidence interval, OR (95% CI), was performed to assess the association of low and middle tertiles of FR versus high FR tertile with mortality risk in subgroups of CKD patients. In all analyses, a P-value < 0.05 was considered statistically significant. The statistical analyses were conducted with Stata Now 18.5 (Stata Corporation, College Station, TX, USA) and Statistical Analysis Systems (SAS) SAS 9.4 level 1 M8 (SAS Campus Drive, Cary, NC, USA).

## Results

### Demographic and clinical characteristics

Among 328 (198 males; 60.4%) patients with a median (IQR) age of 54 (44–63) years and eGFR of 6.3 (4.9–7.9) ml/min/1.73 m^2^, 111 (33.8%) patients had CVD, 104 (31.7%) had DM and 103 (32.7%) were malnourished. Other demographic and clinical characteristics and plasma concentrations of BCAA and AAA and FR are presented in Table 1.

**Table 1:** Demographic and clinical characteristics of 328 CKD stage 5 patients, and comparisons between those in the high tertile and those in the combined middle and low tertiles of Fischer’s ratio.

	All patients	Middle and low tertiles	High tertile	*p*-value
Number	328	217	111	
**Age**,** years**	**54 (44–63)**	**55 (47–63)**	**50 (38–64)**	**0.038**
Female sex, n (%)	130 (39.6%)	94 (43.3%)	36 (32.4%)	0.057
DM, n (%)	104 (31.7%)	66 (30.4%)	38 (34.2%)	0.48
CVD, n (%)	111 (33.8%)	76 (35.0%)	35 (31.5%)	0.53
**PEW**,** n (%)**	**103 (32.7%)**	**78 (37.5%)**	**25 (23.4%)**	**0.011**
BMI, kg/m^2^	24.1 (21.7–27.3)	24.4 (21.9–27.3)	23.6 (21.3–27.4)	0.24
SBP, mm Hg	149 (135–166)	148 (132–164)	150 (139–168)	0.24
DBP, mm Hg	88 (79–97)	87 (77–97)	89 (81–97)	0.38
Pulse pressure, mm Hg	60 (50–74)	60 (48–72)	60 (50–75)	0.56
eGFR, ml/min/1.73·m^2^	6.3 (4.9–7.9)	6.2 (4.7–7.9)	6.4 (5.3-8.0)	0.42
**Triglyceride**,** mmol/L**	**1.9 (1.3–2.5)**	**1.8 (1.3–2.2)**	**2.2 (1.4-3.0)**	**< 0.001**
T-chol, mmol/L	5.2 (4.4–6.3)	5.3 (4.4–6.4)	5.2 (4.2–6.3)	0.80
HDL-c, mmol/L	1.2 (0.9–1.5)	1.2 (0.9–1.6)	1.1 (0.9–1.5)	0.33
**AIP**,** ratio**	**0.4 (0.0–1.0)**	**0.3 (-0.0-0.8)**	**0.6 (0.1–1.2)**	**0.011**
Apo_A, g/L	1.3 (1.1–1.5)	1.3 (1.1–1.5)	1.3 (1.1–1.5)	0.49
Apo_B, g/L	1.0 (0.8–1.3)	1.1 (0.8–1.3)	1.0 (0.8–1.3)	0.88
Lp(a), nmol/L	213 (91–499)	209 (95–450)	228 (64–561)	0.84
Hs-CRP, mg/L	5.0 (2.0–15.0)	5.8 (2.1–16.0)	4.2 (1.6–13.0)	0.071
**IL-6**,** pg/mL**	**6.4 (3.5–10.9)**	**6.7 (3.7–12.8)**	**6.0 (3.3–8.4)**	**0.017**
PTH, pg/mL	206 (93–350)	202 (93–348)	207 (87–355)	0.83
Albumin, g/L	33.0 (29.5–37.5)	33.0 (29.0–38.0)	34.0 (30.0–37.0)	0.61
Hb, g/dL	103 (94–114)	103 (93–114)	103 (96–114)	0.45
**BCAA**,** µmol/L**	**281 (232–329)**	**263 (227–301)**	**318 (273–364)**	**< 0.001**
**AAA**,** µmol/L**	**112 (94–129)**	**117 (102–140)**	**98 (84–113)**	**< 0.001**
**Fischer ratio**	**3.1 (2.7–3.6)**	**2.8 (2.5–3.1)**	**3.8 (3.6–4.3)**	**< 0.001**

Data are expressed as median (interquartile range (IQR)) or numbers (%).

DM, diabetes mellitus; CVD, cardiovascular diseases; PEW, protein energy wasting; BMI, body mass index; SBP, systolic blood pressure; DBP, diastolic blood pressure; TG, triglycerides; T-chol, total cholesterol; HDL, high-density lipoprotein; AIP, atherogenic index of plasma; Apo A, Apolipoprotein A; Apo B, Apolipoprotein B; Lp(a), Lipoprotein (a); Hs-CRP, high-sensitivity C-reactive protein; IL-6, interleukin-6; PTH, parathyroid hormone; Hb, hemoglobin; eGFR, estimated glomerular filtration rate; BCAA, branched-chain amino acid; AAA,​ aromatic amino acid. Bold font indicates significant differences.

### Plasma amino acid concentrations and Fischer´s ratio in patients and healthy controls

**Fig. 1:**
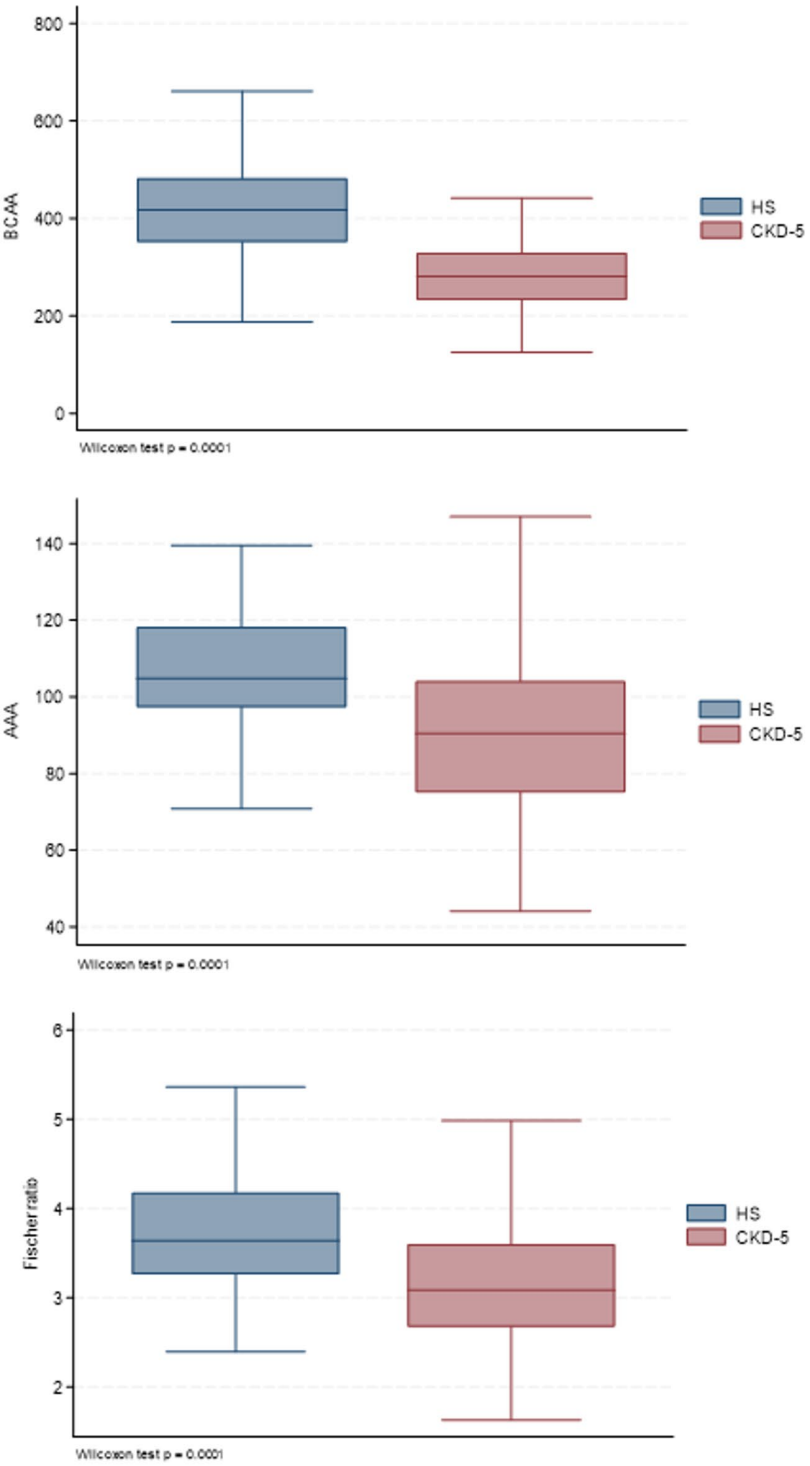
Plasma concentrations of branched-chain amino acids (BCAA), aromatic amino acids (AAA) and Fischer ratio in 328 chronic kidney disease stage 5 (CKD-5) patients and 83 control subjects (HS).

Compared to community-dwelling healthy controls (Fig. 1), plasma concentrations of BCAA (Fig. 1A; 391 (343–459) µmol/l vs. 280 (232–329) µmol/l, *p* < 0001) and AAA (Fig. 1B; 106 (98–119) µmol vs. 90 (75–104) µmol/l, *p* < 0.0001) and FR (Fig. 1C; 3.58 (3.06–4.19) vs. 3.08 (2.68–3.60), *p* < 0.0001) were significantly lower in CKD patients.

FR did not differ significantly (p = 0.8) between those who had started dialysis vs. those who had not yet started dialysis (Supplemental Fig. 1).

Among sixty-one (21.3%) CKD patients receiving oral amino acid supplementation at baseline, plasma FR did not differ between those on supplementation compared to those who were not (3.07 (2.71–3.63) vs. 3.09 (2.64–3.62), *p* = 0.89).

### Comparison between surviving and deceased CKD patients

Compared to surviving patients, the deceased patients were older, had higher systolic and lower diastolic blood pressure, more often CVD, DM and PEW, and had higher hsCRP and IL-6, and lower s-albumin levels (Supplemental Table 1). Patients who died had lower FR compared to survivors (2.9 vs. 3.1, *p* = 0.034), while BCAAs and AAAs were similar.

### Demographic and clinical characteristics among groups with different FR tertiles

The CKD patients were divided into a high FR tertile group and a combined middle plus low FR tertile group (Table 1). Patients in the high FR tertile exhibited lower prevalence of PEW and reduced levels of IL-6, higher triglyceride levels and higher AIP while no significant differences were observed in terms of age, gender, blood pressure, CVD, DM, BMI, s-albumin, hemoglobulin, eGFR or the proportion of patients receiving amino acid supplementation.

Univariate analysis showed significant associations between FR and age, gender, IL-6, and PEW (assessed by SGA) (Supplemental Fig. 2a). In multivariate analysis only age was statistically associated with FR (Supplemental Fig. 2b) and following standardization and converting values to continuous variables, beta values for age, gender and HDL-cholesterol were significantly associated with FR (Supplemental Fig. 2c).

Among 61 patients who received oral amino acid supplementation, 43 (70.5%.) were in the low and middle FR tertile group and 18 (29.5%) in the high FR tertile (*p* = 0.34). The proportion who died or were alive did not differ (22.2% vs. 18.6%; *p* = 0.52).

### Prognostic value of FR tertiles for predicting deaths in CKD patients

**Fig. 2:**
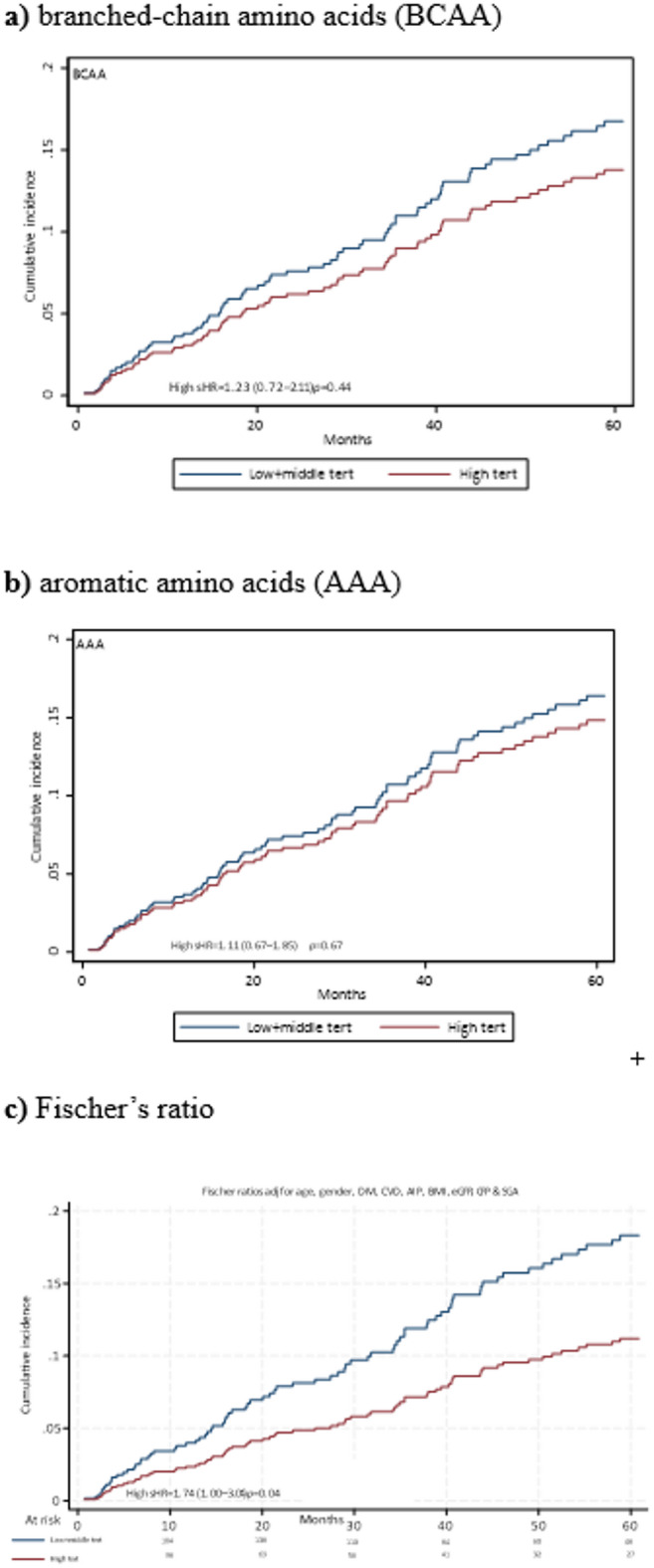
Fine-Gray competing-risk regression models comparing the differences in cumulative mortality in 328 CKD stage 5 patients between the combined middle and low tertiles versus high tertile of (**a**) branched-chain amino acids (BCAA), (**b**) aromatic amino acids (AAA), and (**c**) Fischer’s ratio. Inserts show sub-distribution hazard ratios (sHRs) with 95% confidence intervals. All models were adjusted for age, gender, DM, CVD, eGFR, AIP, PEW, BMI, SGA and CRP. The number of individuals at risk at different time points are shown in Fig. 2**c**.

Fine-Gray competing-risk regression models were used to assess cumulative all-cause mortality risk (expressed as sHR with 95% CI) associated with combined low and middle tertiles versus high tertile of BCAA (Fig. 2a), AAA (Fig. 2b), and FR (Fig. 2c) respectively, after adjustments for age, gender, DM, CVD, eGFR, AIP, SGA (as a marker of PEW), BMI, and CRP (as a marker of inflammation). The results showed that CKD patients in the lower and middle FR tertiles had 74% higher risk of all-cause mortality compared to those in the highest tertile group (sHR 1.74, 95% CI 1.00–3.04, *p* = 0.04; Fig. 2c). In contrast, no such significant differences were observed for BCAA (Fig. 2a) and AAA (Fig. 2b).

Analysis of the associations between 1-SD increase of BCAA, AAA and FR and mortality showed significantly decreased mortality risk of 1-SD increase of FR, sHR 0.75 (95% CI 0.59–0.95); *p* = 0.02 and BCAA, sHR 0.68 (95% CI 0.59–0.89); *p* = 0.006, respectively, but not of AAA, sHR 0.94 (95% CI 0.69–1.24); *p* = 0.69 (Supplemental Fig. 3).

Within six months following dialysis initiation, there were 24 out of a total of 82 deaths: When excluding these early deaths, FR was still associated with increased mortality risk, sHR 1.76 (95% CI 0.97–3.17), *p* = 0.05 (Supplemental Fig. 4).

**Fig. 3:**
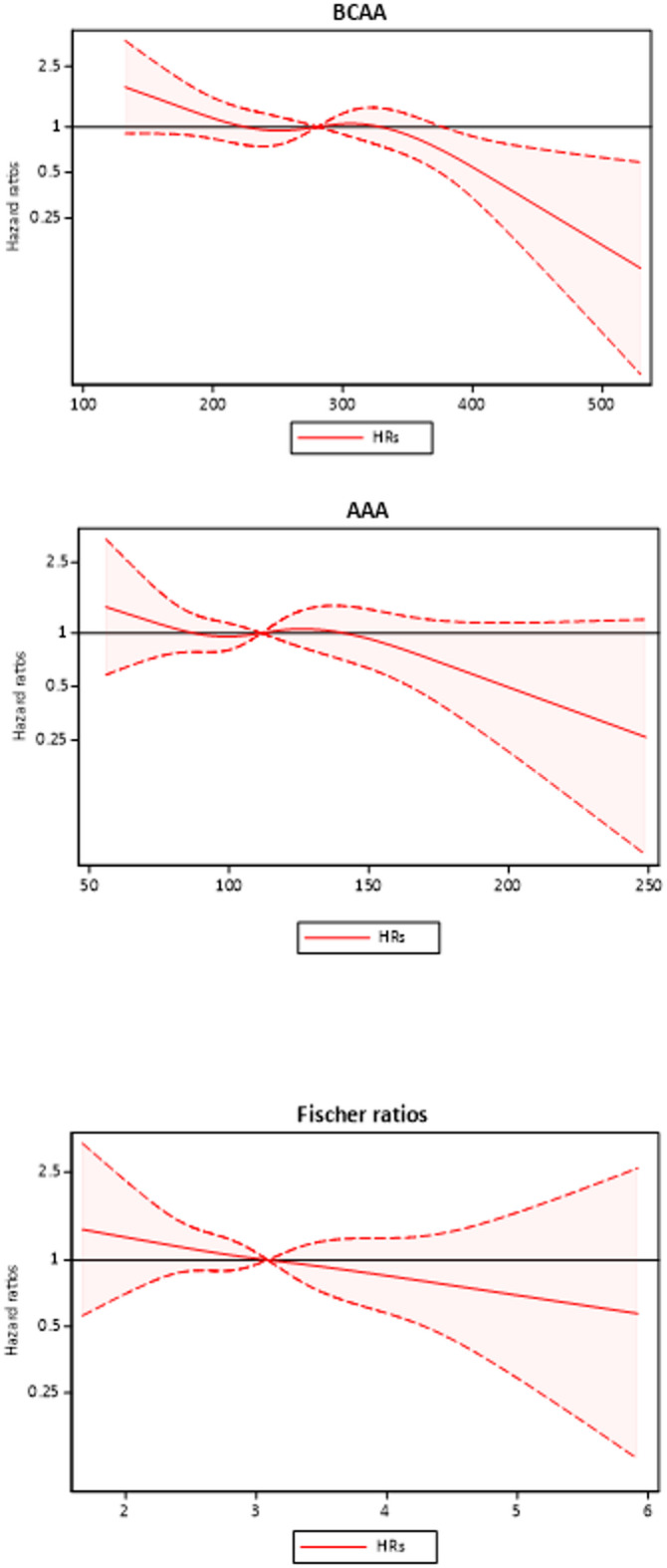
Restricted cubic spline curve of the associations of concentrations (μmol/L) of BCAA and AAA, and the values of FR, as continuous variables, with the subsequent 5-year mortality risk, expressed as HRs and 95% CI in 328 CKD patients, adjusted for age, gender, DM, CVD, hsCRP, eGFR, AIP, PEW and BMI.

In further analysis using restricted cubic spline curves (Fig. 3), inverse linear relationships were observed between FR, BCAA, and AAA, as continuous variables, and the risk of 5-year mortality.

### Subgroup and interaction analysis

**Fig. 4:**
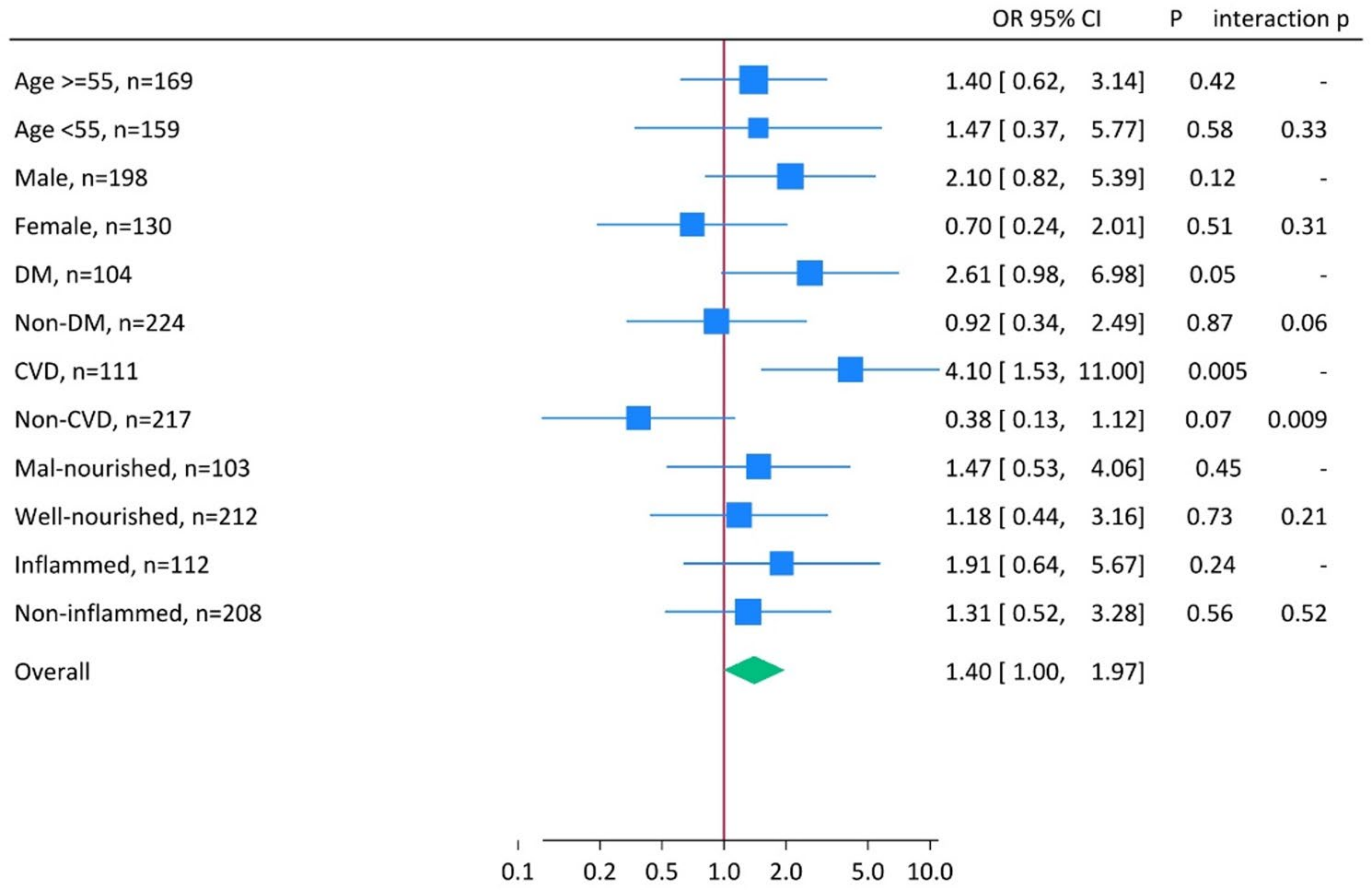
Forest plot of subgroup and interaction analyses for the association between Fischer’s ratio (FR) tertiles and mortality in 328 CKD stage 5 patients starting dialysis. The high FR tertile served as reference versus the combined low and middle tertiles. Multivariate logistic regression analysis yielding odds ratios with 95% confidence interval, OR (95% CI), was performed to assess the association of low and middle tertiles of FR versus high FR tertile with mortality risk in subgroups of CKD patients (n = number of individuals in each subgroup). The size of the squares reflects the precision or weight of each estimate. Definitions of subgroups: Age ≥ 55 vs. < 55 years used as cut off the median age (55 years) of the combined group of middle and low tertiles of FR; DM, diabetes mellitus; CVD, cardiovascular disease; Malnourished vs. well-nourished denotes protein energy wasting (PEW) vs. no PEW, PEW was defined as SGA score > 1; Inflamed vs. non-inflamed denotes presence of inflammation defined according to median hs-CRP of the patients, hs-CRP ≥ 5.0 mg/L vs. hs-CRP < 5.0 mg/L.

Subgroup and interaction analyses (Fig. 4) demonstrated that the association between FR and mortality was not consistent across all patient groups. Compared with patients in the highest FR tertile, those in the combined low and middle tertiles had significantly higher mortality risk in the presence of CVD (OR 4.10, 95% CI 1.53–11.00, *p* = 0.005), with a statistically significant interaction between CVD and non-CVD subgroups (*p* for interaction = 0.009). A borderline significant interaction was also observed for DM versus non-DM (*p* for interaction = 0.06), with a higher risk of death seen in patients with DM (OR 2.61, 95% CI 0.98–6.98, *p* = 0.05). By contrast, no significant effect modification was detected by age, sex, nutritional status, or inflammation. Overall, patients in the combined low and middle FR tertiles had a 1.40-fold increased risk of mortality (95% CI 1.00–1.97).

## Discussion

Kidney failure patients starting dialysis had compared to community-dwelling controls, lower plasma BCAA and AAA concentrations, and a lower FR. Among the patients, a lower FR was independently associated with increased mortality risk, and this appeared to be largely driven by lower BCAA concentrations whereas AAA did not predict mortality. To our knowledge, this is the first study that evaluates the prognostic value of FR in ESRD patients.

In previous studies, FR was used as an indicator to assess the imbalance of amino acids in liver cirrhosis, heart failure, and cancer^27,35^. In ESRD patients, a change in FR was also reported. Małgorzewicz et al.^36^ found a decrease of the BCAAs to AAAs ratio in dialysis patients compared to healthy controls, with further decline in those on dialysis for over two years^36^. This reduction was attributed primarily to decreased BCAA levels and increased AAAs in CKD patients. Several studies report on the reduction of blood BCAA levels in CKD patients^37^. In the present study, BCAA, AAA, and FR were lower than in the controls. FR did not differ significantly between those who were investigated before and after dialysis initiation.

BCAAs are essential amino acids obtained through dietary intake and play key roles in protein synthesis, inhibition of protein degradation, and promotion of skeletal muscle formation^38,39^. Unlike many other amino acids, BCAAs are predominantly metabolized in muscle tissue rather than in the liver and undergo initial enzymatic reactions catalyzed by branched-chain aminotransferase (BCAT) and branched-chain α-ketoacid dehydrogenase (BCKDH). In ESRD patients, there are several pathophysiological conditions that may contribute to the decrease of plasma BCAAs. Uremic toxins can reduce the appetite and decrease dietary intake^39^, and metabolic acidosis enhances BCKDH activity, and eventually accelerates the catabolism of BCAAs^40^. Furthermore, inflammation, PEW and the dialysis process can - through various mechanisms - further disrupt BCAA metabolism and reduce plasma BCAA concentrations in ESRD^18,25,41^.

In contrast, the AAAs are mainly metabolized in the liver^38,39^. The main components of AAAs that were included in the calculation of FR are phenylalanine and tyrosine which are the precursors of catecholamine neurotransmitters and certain uremic toxins^42^, and tyrosine also contributes to thyroid hormone synthesis^43^. Phenylalanine is an essential amino acid, while tyrosine is considered as a conditionally essential amino acid, being synthesized from phenylalanine^23^. In renal failure, impaired conversion from phenylalanine to tyrosine results in an elevated plasma phenylalanine to tyrosine ratio^44^. In addition, oxidative stress and decreased renal excretion contribute to increased tyrosine levels, complicating AAAs metabolism in ESRD^44^.

Importantly, in our study, when comparing patients in the combined low and middle FR tertiles with the high tertile group, FR demonstrated a prognostic effect on overall mortality with low FR indicating higher mortality risk, while neither plasma BCAAs nor AAAs alone had a predictive value. Specifically, the results showed that CKD patients in the lower and middle tertiles of FR had 1.74 times higher risk of all-cause mortality compared to those in the highest tertile. This finding may suggest that the metabolic interactions affecting amino acid profiles in CKD are complex and cannot be explained by changes in a single isolated metabolic pathway alone. Rather, FR may serve as an integrated biomarker, reflecting the balance between catabolic and anabolic processes across organ systems. Specifically, an increase of AAAs relative to BCAAs may indicate impaired hepatic clearance and heightened systemic inflammation, while reduced BCAA availability points to diminished muscle anabolic capacity, inadequate protein intake and progression of protein-energy wasting^45,46^. By capturing both hepatic and muscular metabolic dysfunction, FR provides a dual reflection of systemic metabolic stress, which may explain why it may outperform BCAAs and AAAs as a prognostic marker. On the other hand, when evaluating restricted cubic spline curves of the associations of BCAAs, AAAs, and FR, as continuous variables, with the 5-year mortality risk (Fig. 3), it is evident that the association of high FR with low mortality risk is mainly due to the impact of low BCAAs. Notably, previous studies in non-renal populations have yielded inconsistent results, highlighting the need for further validation in CKD-specific cohorts. A meta-analysis of 10 studies found no association between BCAAs and mortality^47^, while Fung et al.^48^ reported a U-shaped relationship in older adults with hypertension and/or diabetes.

Risk factors for deaths in ESRD patients include both traditional risk factors including high age, CVD and DM (whereas the impact of dyslipidemia is less clear) and non-traditional risk factors such as inflammation, PEW, and anemia, and other factors^15^. In this cohort, deceased patients were older, and exhibited higher frequencies of CVD, DM, smoking, PEW, lower albumin level, and elevated levels of hsCRP and IL-6, findings which are consistent with previous literature.

Notably, we could only identify a few factors (in addition to BCAA and AAA) that were associated negatively with FR, namely age, male sex, IL-6 and PEW. PEW is a potent predictor for mortality in dialyzed and non-dialyzed patients. It has been reported that PEW was associated with increased risk of death in patients with CKD^49^. On one hand, PEW can impair enzyme function related to BCAAs resulting in reduced BCAAs and thereby lower FR^50^. On the other hand, BCAAs, especially leucine, can activate the mTOR signaling pathway and stimulate protein synthesis^38,39^. Thus, the observed alterations in FR may partly reflect the high prevalence of PEW in this cohort.

Inflammation is also common and powerful predictor of mortality in CKD patients and associated with alterations in plasma amino acids profile^18,51^, partly because immune cells rely on amino acids for synthesis of protein. Amino acid imbalance may, in turn, modulate inflammatory pathways^52^. Yoshikawa et al. reported that impairment of lymphocyte function associated with decreased FR with active pulmonary tuberculosis^53^. It is not known whether amino acid imbalances contribute to immune dysfunction and increased susceptibility to infection, a major cause of death among patients with kidney failure^54^. In our study, FR was inversely correlated with IL-6, a reliable biomarker of systemic inflammation and a strong predictor for poor outcomes in CKD patients^55^. Inflammation can also contribute to muscle loss and weakness^56^, further exacerbating nutritional deterioration. Since BCAAs support muscle maintenance and strength, our findings suggest that FR may be a sensitive marker linking inflammation, PEW and survival in CKD.

Dyslipidemia is another risk factor for poor prognosis. In this study, an unexpected observation was that the CKD patients in the highest FR tertile—who had better survival—also had higher serum triglycerides and AIP levels. While elevated triglycerides and AIP are typically associated with higher cardiovascular risk in the general population, this paradox may reflect the phenomenon of ‘reverse epidemiology’ observed in CKD patients^57^. Inflammation and PEW, both of which are prevalent in CKD, can suppress lipid levels^58^. In the present study, the median concentration of triglycerides was low in both the middle and low FR tertiles, while the IL-6 levels were elevated, suggesting that PEW and inflammation may have contributed to reduce triglycerides.

At the same time, our subgroup and interaction analyses provide additional insights. While FR correlated with markers of PEW and inflammation, the prognostic association of FR with mortality was not significantly modified by nutritional or inflammatory status. This indicates that although FR is related to these pathways, its predictive value for survival is not solely explained by them. Instead, the mortality risk associated with lower FR was most pronounced in patients with CVD and, to a lesser extent, DM. Both comorbidities are characterized by chronic metabolic stress, systemic inflammation, oxidative damage, and disordered amino acid metabolism, which may amplify the consequences of an unfavorable balance between BCAAs and AAAs. In DM, impaired insulin signaling and muscle protein turnover further limit BCAA availability, while in CVD, increased catabolism and impaired hepatic clearance of AAAs may exacerbate metabolic imbalance. The lack of significant interaction with inflammation or malnutrition suggests that FR captures broader and more integrated metabolic dysfunction than can be explained by these conventional markers alone.

Taken together, these findings support a dual interpretation: FR correlates with PEW and inflammation and a low FR may serve as a surrogate marker of these processes, but its prognostic strength is most evident in patients with high cardiometabolic comorbidity, particularly CVD. This highlights the multifaceted role of FR as a biomarker that integrates signals from hepatic clearance, muscle anabolism, nutritional state, and systemic inflammation, while also identifying high-risk subgroups in CKD who may benefit from closer metabolic monitoring.

To our knowledge, this is the first study to explore the prognostic value of Fischer’s ratio in CKD stage 5 patients. However, there are some limitations that should be considered when interpreting the results. First, this was an observational study precluding conclusions regarding causality. Second, FR was determined at baseline and there was no subsequent FR measurement. Third, because dietary protein intake was not comprehensively assessed, we cannot exclude its potential influence on circulating amino acid levels and on Fischer’s ratio. Fourth, the study was conducted at a single center, and the sample size was limited, which limits its generalizability. The limited sample size reduced the statistical power of the subgroup and interaction analyses, meaning that some potentially meaningful effect modifications could not be fully captured. Future multi-center studies with larger cohorts and repeated measurements are needed to validate the prognostic value of FR in CKD.

## Conclusion

In patients with CKD stage 5, a lower Fischer’s ratio was associated with increased mortality risk after adjusting for potential confounders, highlighting the potential of FR as a prognostic biomarker in kidney failure patients. The weak or insignificant correlations between FR and potential determinants such as age, gender, serum lipids, inflammation and PEW suggest that FR reflects the complex interplay between many yet undefined factors in CKD. The prognostic strength of FR was most evident in patients with high cardiometabolic comorbidity, particularly CVD, underscoring the need for further research to elucidate the mechanistic links between FR and factors linked to CVD in CKD such as nutritional status and inflammation, and to explore whether targeting amino acid imbalances, particularly those reflected by FR, can improve outcomes in this high-risk population.

## Supplementary Information

Below is the link to the electronic supplementary material.

Supplementary Material 1

## Data Availability

The datasets used and/or analysed during the current study are available from the corresponding author on reasonable request. Data described in the manuscript, code book, and analytic code will be made available upon request pending application to the corresponding author.

## References

[1] Bikbov, B. et al. Global, regional, and national burden of chronic kidney disease, 1990–2017: a systematic analysis for the Global Burden of Disease Study 2017. *Lancet ***395**(10225), 709–733 (2020). 32061315 10.1016/S0140-6736(20)30045-3 PMC7049905

[2] Ferrari, A. J. et al. Global incidence, prevalence, years lived with disability (YLDs), disability-adjusted life-years (DALYs), and healthy life expectancy (HALE) for 371 diseases and injuries in 204 countries and territories and 811 subnational locations, 1990–2021: a systematic analysis for the Global Burden of Disease Study 2021. *Lancet* **403** (10440), 2133–2161 (2024). 38642570 10.1016/S0140-6736(24)00757-8 PMC11122111

[3] Wang, L. et al. Prevalence of chronic kidney disease in China: results from the sixth China chronic disease and risk factor surveillance. *JAMA Intern. Med.* **183** (4), 298–310 (2023). 36804760 10.1001/jamainternmed.2022.6817 PMC9941971

[4] Li, Y. et al. Temporal trends in prevalence and mortality for chronic kidney disease in China from 1990 to 2019: an analysis of the Global Burden of Disease Study 2019. *Clin. Kidney J.* **16** (2), 312–321 (2023). 36755850 10.1093/ckj/sfac218 PMC9900593

[5] United States Renal Data System. *2023 USRDS annual data report: Chronic kidney disease in the general population*. National Institutes of Health, National Institute of Diabetes and Digestive and Kidney Diseases, Bethesda, MD. (2023). Available from: https://usrds-adr.niddk.nih.gov/2023/chronic-kidney-disease

[6] Msaad, R. et al. Predictors of mortality in hemodialysis patients. *Pan Afr. Med. J.* **33**(1), 61 (2019). 10.11604/pamj.2019.33.61.18083 PMC6689835 31448023

[7] Modi, Z. J. et al. Risk of cardiovascular disease and mortality in young adults with end-stage renal disease: an analysis of the US renal data system. *JAMA Cardiol.* **4** (4), 353–362 (2019). 30892557 10.1001/jamacardio.2019.0375 PMC6484951

[8] Gotta, V., Tancev, G., Marsenic, O., Vogt, J. E. & Pfister, M. Identifying key predictors of mortality in young patients on chronic haemodialysis—a machine learning approach. *Nephrol. Dialysis Transplantation*. **36** (3), 519–528 (2021). 10.1093/ndt/gfaa128 32510143

[9] Mallamaci, F. & Tripepi, G. Risk factors of chronic kidney disease progression: between old and new concepts. *J. Clin. Med.* **13** (3), 678 (2024). 38337372 10.3390/jcm13030678 PMC10856768

[10] Orlandi, P. F. et al. A collaborative, individual-level analysis compared longitudinal outcomes across the International Network of Chronic Kidney Disease (iNETCKD) cohorts. *Kidney Int.* **96** (5), 1217–1233 (2019). 31570197 10.1016/j.kint.2019.07.024

[11] Hansrivijit, P. et al. Prediction of mortality among patients with chronic kidney disease: A systematic review. *World J. Nephrol.* **10** (4), 59–75 (2021). 34430385 10.5527/wjn.v10.i4.59 PMC8353601

[12] Mallamaci, F., Tripepi, G., Cutrupi, S., Malatino, L. S. & Zoccali, C. Prognostic value of combined use of biomarkers of inflammation, endothelial dysfunction, and myocardiopathy in patients with ESRD. *Kidney Int.* **67** (6), 2330–2337 (2005). 15882276 10.1111/j.1523-1755.2005.00338.x

[13] Ortiz, A. et al. Clinical usefulness of novel prognostic biomarkers in patients on hemodialysis. *Nat. Rev. Nephrol.* **8** (3), 141–150 (2011). 22045239 10.1038/nrneph.2011.170

[14] Artunc, F. et al. Mortality prediction using modern peptide biomarkers in hemodialysis patients–a comparative analysis. *Kidney Blood Press. Res.* **39** (6), 563–572 (2014). 25531828 10.1159/000368468

[15] Sun, J. et al. Biomarkers of Cardiovascular Disease and Mortality Risk in Patients with Advanced CKD. *Clin. J. Am. Soc. Nephrol.* **11** (7), 1163–1172 (2016). 27281698 10.2215/CJN.10441015 PMC4934843

[16] Garibotto, G. et al. Amino acid and protein metabolism in the human kidney and in patients with chronic kidney disease. *Clin. Nutr.* **29** (4), 424–433 (2010). 20207454 10.1016/j.clnu.2010.02.005

[17] Bergström, J., Alvestrand, A. & Fürst, P. Plasma and muscle free amino acids in maintenance hemodialysis patients without protein malnutrition. *Kidney Int.* **38** (1), 108–114 (1990). 2117095 10.1038/ki.1990.174

[18] Suliman, M. E. et al. Inflammation contributes to low plasma amino acid concentrations in patients with chronic kidney disease. *Am. J. Clin. Nutr.* **82**(2), 342–349 (2005). 16087977 10.1093/ajcn.82.2.342

[19] Divino Filho, J., Barany, P., Stehle, P., Fürst, P. & Bergström, J. Free amino-acid levels simultaneously collected in plasma, muscle, and erythrocytes of uraemic patients. *Nephrol Dial Transplant. ***12**(11), 2339–2348 (1997). 9394321 10.1093/ndt/12.11.2339

[20] Knol, M. G., Wulfmeyer, V. C., Müller, R-U. & Rinschen, M. M. Amino acid metabolism in kidney health and disease. *Nat. Rev. Nephrol.* **20** (12), 771–788 (2024). 39198707 10.1038/s41581-024-00872-8

[21] Li, X., Zheng, S. & Wu, G. Amino acid metabolism in the kidneys: nutritional and physiological significance. Adv Exp Med Biol. **1265**, 71–95 (2020). 10.1007/978-3-030-45328-2_5 32761571

[22] Duranton, F. et al. Plasma and urinary amino acid metabolomic profiling in patients with different levels of kidney function. *Clin. J. Am. Soc. Nephrol.* **9** (1), 37–45 (2014). 24235289 10.2215/CJN.06000613 PMC3878706

[23] Liu, L. et al. Metabolic homeostasis of amino acids and diabetic kidney disease. *Nutrients* **15** (1), 184 (2022). 36615841 10.3390/nu15010184 PMC9823842

[24] Soeters, P. & Fischer, J. Insulin, glucagon, aminoacid imbalance, and hepatic encephalopathy. *Lancet* **308** (7991), 880–882 (1976). 10.1016/s0140-6736(76)90541-9 62115

[25] Gervasini G. et al. Prognostic Significance of Amino Acid and Biogenic Amines Profiling in Chronic Kidney Disease. *Biomedicines.* **11**(10), 2775 (2023). 10.3390/biomedicines11102775 PMC10604890 37893147

[26] Ghanem, S. E. et al. Evaluation of amino acids profile as non-invasive biomarkers of hepatocellular carcinoma in Egyptians. *Trop. Med. Infect. Disease*. **7** (12), 437 (2022). 36548692 10.3390/tropicalmed7120437 PMC9786038

[27] Hiraiwa, H. et al. Usefulness of the plasma branched-chain amino acid/aromatic amino acid ratio for predicting future cardiac events in patients with heart failure. *J. Cardiol.* **75** (6), 689–696 (2020). 32001073 10.1016/j.jjcc.2019.12.016

[28] Tajiri, K. & Shimizu, Y. Branched-chain amino acids in liver diseases. *Translational Gastroenterol. Hepatol.* **3**, 47 (2018). 10.21037/tgh.2018.07.06 PMC6088198 30148232

[29] Stenvinkel, P. et al. Strong association between malnutrition, inflammation, and atherosclerosis in chronic renal failure. *Kidney Int.* **55** (5), 1899–1911 (1999). 10231453 10.1046/j.1523-1755.1999.00422.x

[30] Mukai, H. et al. Restrictive lung disorder is common in patients with kidney failure and associates with protein-energy wasting, inflammation and cardiovascular disease. *PLoS One*. **13** (4), e0195585 (2018). 29702682 10.1371/journal.pone.0195585 PMC5922538

[31] Ghanavatian, S. et al. Subclinical atherosclerosis, endothelial function, and serum inflammatory markers in chronic kidney disease stages 3 to 4. *Angiology* **65** (5), 443–449 (2014). 23567479 10.1177/0003319713483000

[32] Suliman, M. E., Anderstam, B. & Bergström, J. Evidence of taurine depletion and accumulation of cysteinesulfinic acid in chronic dialysis patients. *Kidney Int.* **50** (5), 1713–1717 (1996). 8914041 10.1038/ki.1996.490

[33] Dobiasova, M. & Frohlich, J. The new atherogenic plasma index reflects the triglyceride and HDL-cholesterol ratio, the lipoprotein particle size and the cholesterol esterification rate: changes during lipanor therapy. *Vnitr. Lek.* **46** (3), 152–156 (2000). 11048517

[34] Qureshi, A. R. et al. Factors predicting malnutrition in hemodialysis patients: a cross-sectional study. *Kidney Int.* **53** (3), 773–782 (1998). 9507226 10.1046/j.1523-1755.1998.00812.x

[35] Jimenez Jimenez, F., Ortiz Leyba, C., Morales Mendez, S., Barros Perez, M. & Munoz Garcia, J. Prospective study on the efficacy of branched-chain amino acids in septic patients. *J. Parenter. Enter. Nutr.* **15** (3), 252–261 (1991). 10.1177/0148607191015003252 1907675

[36] Małgorzewicz, S., Dębska-Ślizień, A., Rutkowski, B. & Łysiak-Szydłowska, W. Serum concentration of amino acids versus nutritional status in hemodialysis patients. *J. Ren. Nutr.* **18** (2), 239–247 (2008). 18267217 10.1053/j.jrn.2007.11.011

[37] Cano, N. J. Branched-chain amino-acid metabolism in renal failure. *J. Ren. Nutr.* **19** (5 Suppl), S22–S24 (2009). 19712871 10.1053/j.jrn.2009.06.014

[38] Zhang, S., Zeng, X., Ren, M., Mao, X. & Qiao, S. Novel metabolic and physiological functions of branched chain amino acids: a review. *J. Anim. Sci. Biotechnol.* **8**, 1–12 (2017). 28127425 10.1186/s40104-016-0139-z PMC5260006

[39] Holeček, M. Branched-chain amino acids in health and disease: metabolism, alterations in blood plasma, and as supplements. *Nutr. Metab (Lond)*. **15**, 33 (2018) 10.1186/s12986-018-0271-1 PMC5934885 29755574

[40] Dahabiyeh, L. A. et al. Metabolomics profiling distinctively identified end-stage renal disease patients from chronic kidney disease patients. *Sci. Rep.* **13** (1), 6161 (2023). 37061630 10.1038/s41598-023-33377-8 PMC10105740

[41] Kopple, J. D., Jones, M., Fukuda, S. & Swendseid, M. E. Amino acid and protein metabolism in renal failure. *Am. J. Clin. Nutr.* **31** (9), 1532–1540 (1978). 685869 10.1093/ajcn/31.9.1532

[42] Barba, C. et al. A low aromatic amino-acid diet improves renal function and prevent kidney fibrosis in mice with chronic kidney disease. *Sci. Rep.* **11** (1), 19184 (2021). 34584168 10.1038/s41598-021-98718-x PMC8479128

[43] Stančíková, M., Rovenský, J. Metabolism of Aromatic Amino Acids. p. 9–12 In: Rovenský, J., Urbánek, T., Oľga, B., Gallagher, J. (eds) *Alkaptonuria and Ochronosis*. Springer, Cham. (2015)

[44] Kopple, J. D. Phenylalanine and tyrosine metabolism in chronic kidney failure. *J. Nutr.* **137** (6 Suppl 1):1586S-1590S (2007). 17513431 10.1093/jn/137.6.1586S

[45] Nie, C., He, T., Zhang, W., Zhang, G. & Ma, X. Branched chain amino acids: beyond nutrition metabolism. *Int. J. Mol. Sci.* **19** (4), 954 (2018). 29570613 10.3390/ijms19040954 PMC5979320

[46] Dejong, C. H., van de Poll, M. C., Soeters, P. B., Jalan, R. & Damink, S. W. O. Aromatic amino acid metabolism during liver failure. *J. Nutr.* **137** (6 Suppl 1):1579S-1585S (2007). 17513430 10.1093/jn/137.6.1579S

[47] Teymoori, F. et al. Serum branched amino acids and the risk of all-cause mortality: a meta-analysis and systematic review. *Amino Acids*. **55** (11), 1475–1486 (2023). 37725184 10.1007/s00726-023-03329-7

[48] Fung, E. et al. Divergent Survival Outcomes Associated with Elevated Branched-Chain Amino Acid Levels among Older Adults with or without Hypertension and Diabetes: A Validated, Prospective, Longitudinal Follow-Up Study. *Biomolecules* **13** (8), 1252 (2023). 37627317 10.3390/biom13081252 PMC10452866

[49] Rashid, I. et al. Malnutrition as a Potential Predictor of Mortality in Chronic Kidney Disease Patients on Dialysis: A Systematic Review and Meta-Analysis. *Clin. Nutr.* **43**(7), 1760–1769 (2024). 10.1016/j.clnu.2024.05.037 38852509

[50] Dimou, A., Tsimihodimos, V. & Bairaktari, E. The critical role of the branched chain amino acids (BCAAs) catabolism-regulating enzymes, branched-chain aminotransferase (BCAT) and branched-chain α-keto acid dehydrogenase (BCKD), in human pathophysiology. *Int. J. Mol. Sci.* **23** (7), 4022 (2022). 35409380 10.3390/ijms23074022 PMC8999875

[51] Silverstein, D. M. Inflammation in chronic kidney disease: role in the progression of renal and cardiovascular disease. *Pediatr. Nephrol.* **24** (8), 1445–1452 (2009). 19083024 10.1007/s00467-008-1046-0

[52] Tomé, D. Amino acid metabolism and signalling pathways: potential targets in the control of infection and immunity. *Eur. J. Clin. Nutr.* **75** (9), 1319–1327 (2021). 34163018 10.1038/s41430-021-00943-0 PMC8220356

[53] Yoshikawa, M. et al. Relationship between nutritional depletion and cell-mediated immune function in active pulmonary tuberculosis. Kekkaku (Tuberculosis). **69**(4), 307–316 (1994). 8189684

[54] Kato, S. et al. Aspects of immune dysfunction in end-stage renal disease. *Clin. J. Am. Soc. Nephrol.* **3** (5), 1526–1533 (2008). 18701615 10.2215/CJN.00950208 PMC4571158

[55] Zhang, W. et al. Prognostic role of C-reactive protein and interleukin-6 in dialysis patients: a systematic review and meta-analysis. *J. Nephrol.* **26** (2), 243–253 (2013). 22684644 10.5301/jn.5000169

[56] Schaap, L. A., Pluijm, S. M., Deeg, D. J. & Visser, M. Inflammatory markers and loss of muscle mass (sarcopenia) and strength. *Am. J. Med.* **119** (6), 526 (2006). e9–. e17. 10.1016/j.amjmed.2005.10.049 16750969

[57] Kalantar-Zadeh K. What is so bad about reverse epidemiology anyway? *Semin Dial. ***20**(6), 593–601 (2007). 10.1111/j.1525-139X.2007.00360.x 17991210

[58] Chmielewski, M., Carrero, J. J., Nordfors, L., Lindholm, B. & Stenvinkel, P. Lipid disorders in chronic kidney disease: reverse epidemiology and therapeutic approach. *J. Nephrol.* **21** (5), 635–644 (2008). 18949717

