# Supplementary material for: Low Fischer’s ratio is associated with increased mortality in patients with kidney failure
Source: Sci Rep. 2026 Apr 6;16:11603. doi: 10.1038/s41598-026-46326-y (PMC13056974; doi:10.1038/s41598-026-46326-y)
Supplement: Supplementary file 1 — Supplementary Material 1 [file 41598_2026_46326_MOESM1_ESM.pdf]

## Supplemental material

## Tables

**Supplemental Table 1.** Comparisons of demographic and clinical characteristics of 328 CKD stage 5 patients based on five-year survival status (alive vs. deceased).

| | Alive (*n=246)* | Died (*n=82)* | p-value |
|---|---|---|---|
| Age, years | 50 (41-60) | 64 (56-67) | <0.001 |
| Female sex, n (%) | 100 (40.7%) | 30 (36.6%) | 0.51 |
| DM, n (%) | 62 (25.2%) | 42 (51.2%) | <0.001 |
| CVD, n (%) | 59 (24.0%) | 52 (63.4%) | <0.001 |
| PEW, n (%) | 52 (22.0%) | 51 (64.6%) | <0.001 |
| BMI, kg/m$^2$ | 24.0 (21.7-27.4) | 24.2 (21.9-27.3) | 0.94 |
| SBP, mm Hg | 147 (134-162) | 156 (138-175) | 0.026 |
| DBP, mm Hg | 89 (80-98) | 86 (74-94) | 0.046 |
| TG, mmol/L | 1.9 (1.4-2.5) | 1.8 (1.3-2.6) | 0.66 |
| T-chol, mmol/L | 5.3 (4.4-6.3) | 5.1 (4.2-6.6) | 0.66 |
| HDL-c, mmol/L | 1.2 (0.9-1.5) | 1.1 (0.9-1.6) | 0.95 |
| AIP, ratio | 0.4 (0.0-1.0) | 0.4 (0.0-0.9) | 0.86 |
| Apo_A, g/L | 1.3 (1.1-1.5) | 1.3 (1.1-1.5) | 0.41 |
| Apo_B, g/L | 1.1 (0.8-1.3) | 1.0 (0.8-1.3) | 0.83 |
| Lp(a),nmol/L | 195 (79-478) | 279 (97-524) | 0.13 |
| Hs-CRP, mg/L | 4.0 (1.6-13.0) | 12.0 (3.0-26.0) | <0.001 |
| IL-6, pg/mL | 5.7 (3.1-9.5) | 8.8 (5.9-15.1) | <0.001 |
| PTH, pg/mL | 206 (91-350) | 208 (108-370) | 0.76 |
| Albumin, g/L | 34 (31-38) | 30 (26-35) | <0.001 |
| Hb, g/dL | 104 (95-115) | 100 (93-110) | 0.15 |
| eGFR, ml/min/1.73·m$^2$ | 6.3 (4.9-7.8) | 6.3 (4.8-8.2) | 0.46 |
| BCAA, μmol/L | 284 (233-333) | 273 (231-323) | 0.17 |
| AAA, μmol/L | 112 (92-128) | 111 (95-134) | 0.75 |
| Fischer ratio | 3.1 (2.7-3.7) | 2.9 (2.7-3.3) | 0.034 |

Data are expressed as median (interquartile range (IQR)) or numbers (%). Abbreviations: DM, diabetes mellitus; CVD, cardiovascular diseases; PEW, protein energy wasting; BMI, body mass index; SBP, systolic blood pressure; DBP, diastolic blood pressure; TG, Triglyceride; T-chol, total cholesterol; HDL, high-density lipoprotein; AIP, atherogenic index of plasma; Apo A, Apolipoprotein A; Apo B, Apolipoprotein B; Lp(a), Lipoprotein (a); Hs-CRP, high-sensitivity C-reactive protein; IL-6, interleukin-6; PTH, parathyroid hormone; Hb, hemoglobin; eGFR, estimated glomerular filtration rate; BCAA, branched-chain amino acid; AAA, aromatic amino acid.

## Figures

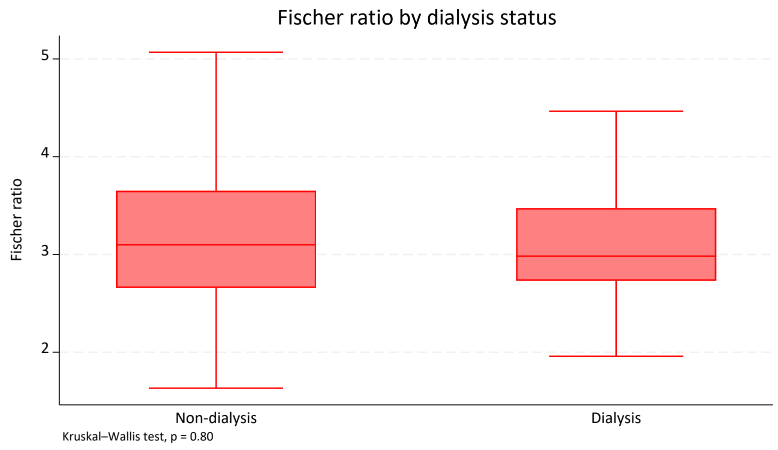


**Supplemental Figure 1.** Fischer ratio by dialysis status, non-dialysis (n=229) vs. dialysis (n=99). In 229 (69.8%) of the patients, the baseline investigation was performed median (IQR) 21(5-77) days before the start of dialysis and in 99 (30.2%) of the patients the baseline investigation was performed within 8(4-21) days after dialysis initiation.

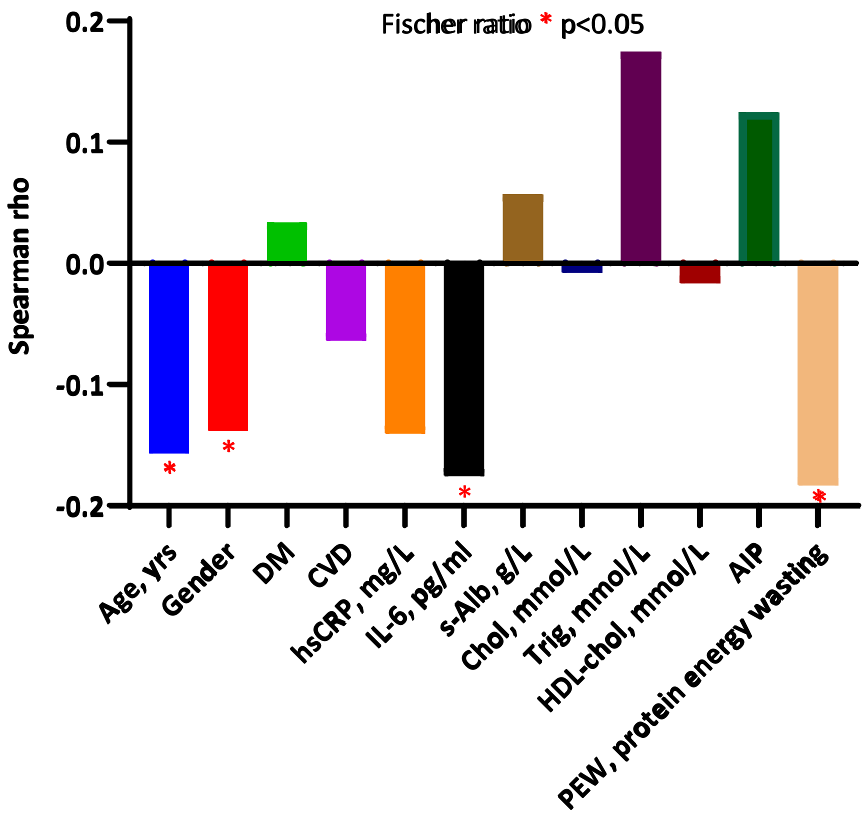


**Supplemental Figure 2a.** Univariate analysis of factors associated with Fischer ratio other than BCAA and AAA (rho, Spearman rank).

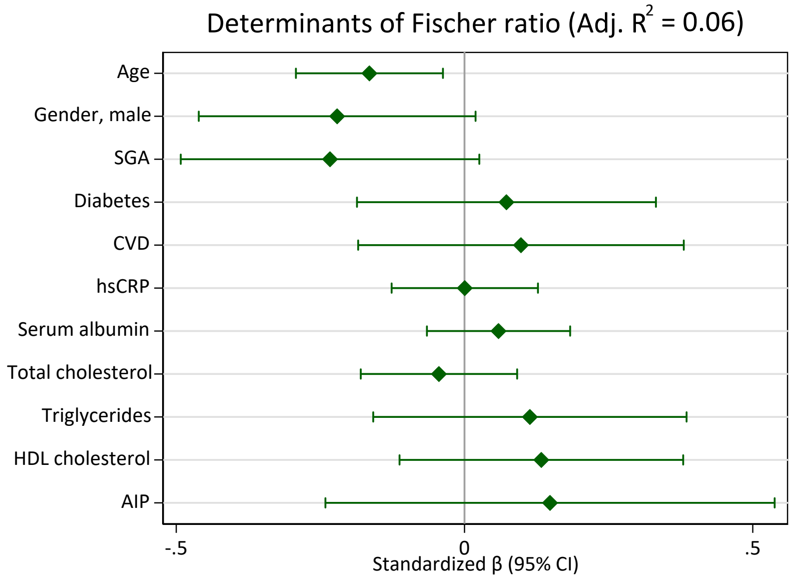


**Supplemental Figure 2b.** Multivariate analysis of factors associated with Fischer ratio expressed as standardized β (95%CI).

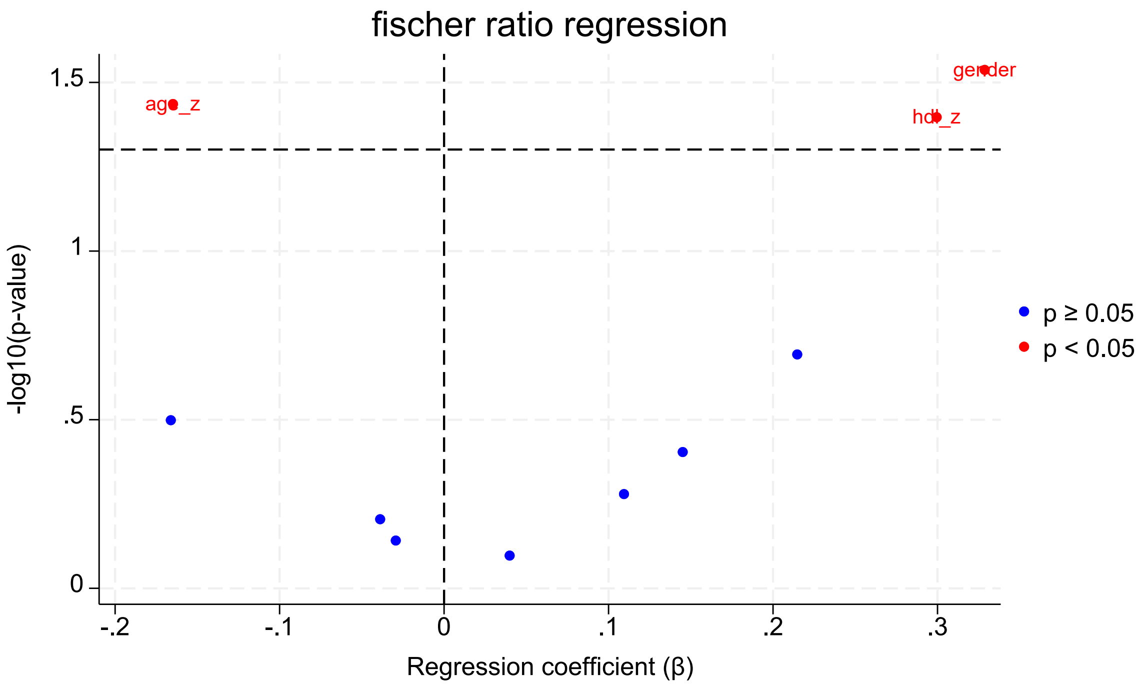


**Supplemental Figure 2c.** Volcano plot of factors associated with Fischer ratio in multivariate analysis. Red and blue dots represent statistically significant or not statistically significant β values. Age, gender and HDL-cholesterol were significantly associated with Fischer ratio.

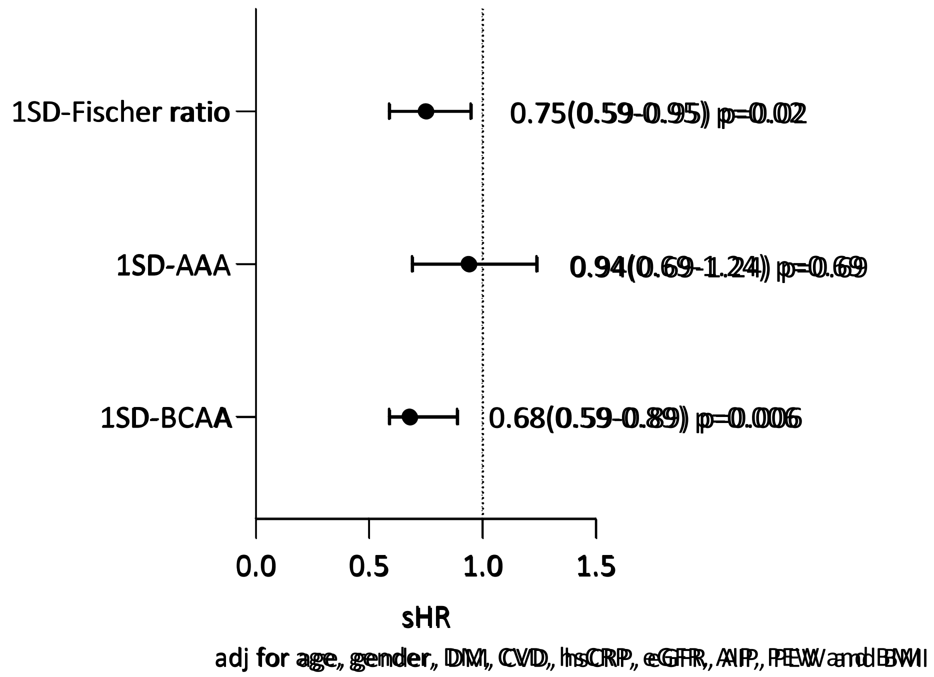


**Supplemental Figure 3.** Associations of 1-SD of AAA, BCAA and FR with sHR (95% CI) of mortality within follow up of 5-years, adjustments for age, gender, DM, CVD, hsCRP, eGFR, AIP, PEW and BMI.

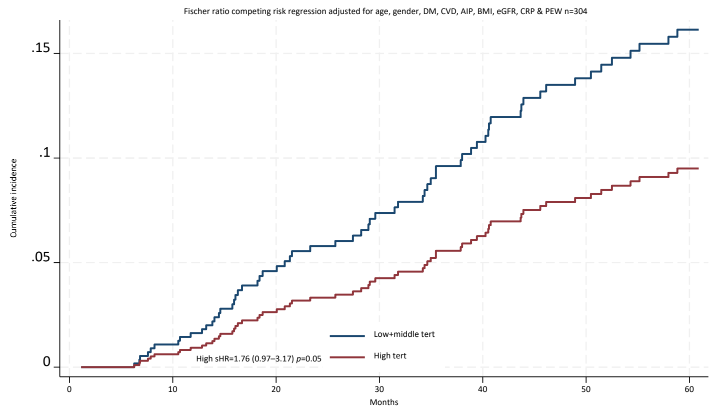


**Supplemental Figure 4**. Fine-Gray competing-risk regression model comparing cumulative mortality in combined middle and low tertiles versus high tertile of Fischer's ratio excluding early deaths occurring within 6 months after the baseline investigation (24 deaths out of a total of 80 deaths during the 60-month follow-up).
